# Work Kills No Man

**Published:** 1890-02

**Authors:** 


### Work Kills no Man.

From the Harveian Oration delivered at the Royal College of Physicians, October 18, 1889, by J. E. Pollack, M.D., F.R.C.P., we quote the following concluding sentences:

I need not say to such an audience as this that *work*—the due exercise of every function given us—kills no man and shortens no life. The causes are to be found in what is called our extended civilization. We are no longer traders to one country, nor for one or two commodities; but the telegraph has introduced us into a widened sphere, and our merchants have investments in every climate, and enter on risks of a kind so varied that the knowledge of no one man is sufficient to grasp it.

Hence there are the anxieties of extended speculation, and a necessary want of the perfect understanding of each. The knowledge of one kind of trade was formerly "power," and led to prosperity; now we are playing games with all the world. Those who are present know well what part of the organism it is which generally fails under such pressure. The public say it is *brain*, but we know that it is *heart*—the motor power which Harvey studied, although, perhaps, he did not foresee to what pressure a modern civilization and struggle would subject it.

I have spoken but of the trading class and the speculative class, but all classes of society should learn to counteract in themselves the depressing agents of excessive worry, and to beware of the race which, once entered on, may exceed the best of our powers and ruin the machine.

